# Management of a large tuberculosis contact investigation related to a contaminated bone graft product used in spinal surgery

**DOI:** 10.1017/ash.2022.165

**Published:** 2022-05-16

**Authors:** Marci Drees, Lija Gireesh, Carol Briody, Charlotte Miller, Emily Hanlin, Ruoran Li, William Wilson, Noah Schwartz, Isaac Benowitz, Janet Glowicz, Tabe Mase

## Abstract

**Background:** In March–April 2021, 23 patients at a 906-bed hospital in Delaware had surgical implantation of a bone graft product contaminated with *Mycobacterium tuberculosis*; 17 patients were rehospitalized for surgical site infections and 6 developed pulmonary tuberculosis. In May 2021, we investigated this tuberculosis outbreak and conducted a large, multidisciplinary, contact investigation among healthcare personnel (HCP) and patients potentially exposed over an extended period in multiple departments. **Methods:** Exposed HCP were those identified by their managers as present, without the use of airborne precautions, in operating rooms (ORs) during index spine surgeries or subsequent procedures, the postanesthesia care unit (PACU) when patients had draining wounds, inpatient rooms when wound care was performed, and the sterile processing department (SPD) on the days repeated surgeries were performed. We created and assigned an online education module and symptom screening questionnaire to exposed HCP. Employee health services (EHS) instituted a dedicated phlebotomy station to provide interferon-γ release assay (IGRA) testing for HCP at ≥8 weeks after last known exposure. EHS managed all exposed HCP, including nonemployees (eg, private surgeons) via automated e-mail reminders, which were escalated through supervisory chains as needed until follow-up completion. The infection prevention team notified exposed patients, defined as those who shared semiprivate rooms with case patients with transmissible tuberculosis. The Delaware Division of Public Health performed IGRA testing. **Results:** There were 506 exposed HCP in ORs (n = 100), the PACU (n = 87), inpatient units (n = 140), the SPD (n = 54), and other locations (n = 122); 83% were employed by the health system. Surgical masks and eye protection were routinely used during patient care. All exposed HCP completed screening by December 17, 2021. Furthermore, 2 HCP had positive IGRAs without symptoms or chest radiograph abnormalities, indicating latent tuberculosis infection, but after further review of records and interviews, we discovered that they had previously tested positive and had been treated for latent tuberculosis infection. In addition, 5 exposed patients tested negative and 2 remain pending. **Conclusions:** This large investigation demonstrated the need for a systematic process that encompassed all exposed HCP including nonemployees and incorporated administrative controls to ensure complete follow-up. We did not identify any conversions related to this outbreak despite high burden of disease in case patients and multiple exposures to contaminated bone-graft material and infectious bodily fluids without respirator use. Transmission risk was likely reduced by baseline surgical mask use and rapid institution of airborne precautions after outbreak recognition.

**Funding:** None

**Disclosures:** None

